# Air Pollution and Postneonatal Infant Mortality in the United States, 1999–2002

**DOI:** 10.1289/ehp.10370

**Published:** 2007-10-24

**Authors:** Tracey J. Woodruff, Lyndsey A. Darrow, Jennifer D. Parker

**Affiliations:** 1 Department of Obstetrics, Gynecology, and Reproductive Sciences, University of California, San Francisco, California, USA; 2 Department of Epidemiology, Emory University, Atlanta, Georgia, USA; 3 National Center for Health Statistics, Centers for Disease Control and Prevention, Hyattsville, Maryland, USA

**Keywords:** carbon monoxide, ozone, particulate matter air pollution, postneonatal infant mortality, respiratory-related deaths, sudden infant death syndrome, sulfur dioxide

## Abstract

**Objective:**

Our goal was to evaluate the relationship between cause-specific postneonatal infant mortality and chronic early-life exposure to particulate matter and gaseous air pollutants across the United States.

**Methods:**

We linked county-specific monitoring data for particles with aerodiameter of ≤ 2.5 μm (PM_2.5_) and ≤ 10 μm (PM_10_), ozone, sulfur dioxide, and carbon monoxide to birth and death records for infants born from 1999 to 2002 in U.S. counties with > 250,000 residents. For each infant, we calculated the average concentration of each pollutant over the first 2 months of life. We used logistic generalized estimating equations to estimate odds ratios of postneonatal mortality for all causes, respiratory causes, sudden infant death syndrome (SIDS), and all other causes for each pollutant, controlling for individual maternal factors (race, marital status, education, age, and primiparity), percentage of county population below poverty, region, birth month, birth year, and other pollutants. This analysis includes about 3.5 million births, with 6,639 postneonatal infant deaths.

**Results:**

After adjustment for demographic and other factors and for other pollutants, we found adjusted odds ratios of 1.16 [95% confidence interval (CI), 1.06–1.27] for a 10-μg/m^3^ increase in PM_10_ for respiratory causes and 1.20 (95% CI, 1.09–1.32) for a 10-ppb increase in ozone and deaths from SIDS. We did not find relationships with other pollutants and for other causes of death (control category).

**Conclusions:**

This study supports particulate matter air pollution being a risk factor for respiratory-related postneonatal mortality and suggests that ozone may be associated with SIDS in the United States.

Several studies have found a relationship between particulate matter (PM) air pollution and infant mortality in countries with relatively high levels of PM air pollution as well as in countries with lower pollution levels, such as Canada and the United States (Bobak and Leon 1999; Ha et al. 2003; Lipfert et al. 2000; Loomis et al. 1999; Ritz et al. 2006; Woodruff et al. 1997, 2006) These studies suggest that PM air pollution is more strongly associated with postneonatal mortality (deaths occurring after 28 days of life) than with neonatal mortality (deaths occurring up to 28 days of life) and that the association with postneonatal mortality appears to be specific to respiratory causes (Bobak and Leon 1999; Ha et al. 2003). However, a number of questions remain about the infant mortality and PM air pollution relationship and the role of other air pollutants as either potential confounders of the relationship or as independent predictors of infant mortality.

Until recently, most studies of air pollution and postneonatal infant mortality have focused on larger particles, either measured as total suspended particles or particles with an aerodiameter of ≤ 10 μm (PM_10_) (Bobak and Leon 1999; Ha et al. 2003; Lipfert et al. 2000; Ritz et al. 2006; Woodruff et al. 1997). Although monitoring of smaller particles measuring ≤ 2.5 μm (PM_2.5_) has become more widespread, only one study in California has evaluated PM_2.5_ in relation to respiratory related infant mortality; results support a positive association (Woodruff et al. 2006). Although many studies in adults suggest that PM_2.5_ is more strongly associated with respiratory and cardiovascular morbidity and mortality than PM_10_, other studies have found larger particles to be important for certain outcomes (Brunekreef and Forsberg 2005; Pope and Dockery 2006).

In addition, few studies have evaluated the contribution of other pollutants to infant mortality, either on their own or as confounders of the association between particles and infant mortality. A study in the Czech Republic, examining nitrogen dioxide and sulfur dioxide, did not find either pollutant significantly associated with postneonatal respiratory deaths after including the other study pollutants in the model (Bobak and Leon 1999). In a study of U.S. infants born in 1990, controlling for carbon monoxide in the regression models did not affect the observed relationship between PM and infant mortality (Lipfert et al. 2000). Another study in Southern California has found some suggestion of an independent effect of CO on respiratory postneonatal infant mortality and an association between NO_2_ and sudden infant death syndrome (SIDS) (Ritz et al. 2006).

Finally, although relatively consistent results have been found for PM and respiratory postneonatal infant mortality, varying results have been found for the association between PM and SIDS. PM_10_ had been found in earlier studies to be associated with SIDS in the United States (Lipfert et al. 2000; Woodruff et al. 1997). However, a study in Canada found that short-term increases in NO_2_ and SO_2_, but not PM_2.5_, were associated with SIDS between 1984 and 1999 (Dales et al. 2004). Similarly, a recent analysis of births in California during 1999–2000 did not find a relationship between PM_2.5_ and SIDS (Woodruff et al. 2006).

Given the uncertainty in findings for different particle size (coarse vs. fine), the varied findings for studies with multiple pollutants, and the variability in the results for SIDS, further evaluation is warranted. In this study, we addressed these issues and evaluated the role of chronic exposure to gaseous air pollutants (CO, SO_2_, and ozone) and different particle size (both PM_2.5_ and PM_10_) in a more contemporary national data set. We used linked infant birth–death records for infants born in the United States between 1999 and 2002 to examine the relationships between these air pollutants and postneonatal respiratory and SIDS infant mortality.

## Methods

### Study population

We obtained linked birth and infant death files from the National Center for Health Statistics, which consist of birth certificate data linked to death certificates for those infants who die within the first year of life, for all births during 1999–2002 (National Center for Health Statistics 2005). Births in counties with < 250,000 residents were not eligible for our study population because these counties are not identified on public-use files. We limited eligible births for our study to singleton births with known birth order, known maternal race, known maternal education, known marital status, known maternal age, known birth weight, and a reported gestational age of up to 44 weeks. Maternal race and Hispanic origin were collapsed into a single categorical variable with three levels: non-Hispanic African American (hereafter black), Hispanic, and non-Hispanic white (hereafter white). Asian and American Indian deaths were excluded from the analyses because of small numbers of infant deaths. We did not include infants who died in the neonatal period (before 28 days) as eligible births because these deaths often happen before the infant leaves the hospital and are often attributed to pregnancy complications or other factors intrinsically related to the infant. Finally, we excluded postneonatal deaths if the residential county at birth did not match the residential county at death. These criteria gave an eligible study population of 7,991,974 births of 16,066,160 births during the study period. The decrease was attributed primarily to the exclusion of births in counties containing < 250,000 residents.

Of the postneonatal deaths in our study population, about 50% of the infants died between 28 days and 3 months of age, about 30% died between 3 and 6 months, and the remaining 20% of infants died between 6 months and 1 year of age; this distribution of age of death was similar to that for all post-neonatal deaths in the United States in 2002 (data not shown).

### Exposure assessment

PM_10_, PM_2.5_, O_3_, CO, and SO_2_ monitoring data for 1999–2002 were obtained from the U.S. Environmental Protection Agency (2004). Both PM_10_ and PM_2.5_ were typically measured continuously for 24 hr once every 6 days. Gaseous pollutant measurements were made daily. For O_3_, we used the 24-hr daily average; long-term averages based on the 24-hr or maximum daily 8-hr average are highly correlated (data not shown). To focus on pollution monitors most likely to reflect population exposures, we excluded monitors intended to capture extreme downwind/upwind pollutant levels, background levels, maximum levels, specific source impacts, and economic impacts.

To calculate chronic exposures for our study population, we matched eligible births with the pollutant data by mother’s county of residence. For each infant, we calculated the average concentration of each pollutant over the first 2 months of life as a measure of chronic exposure. Monthly averages were calculated only if there were at least three available measures for PM and at least 15 available measures for the gaseous pollutants over the course of the month. Infants without air pollution measures for all pollutants for both of the first 2 months of life were excluded. After considering the use of other exposures, the 2-month exposure window was considered appropriate because *a*) a large number of post-neonatal infant deaths occur within 2 months; *b*) we were able to assign comparable exposures to the deaths and the surviving infants; *c*) larger windows of exposure time would have reduced the number of births in the study due to missing data, and a shorter window may not have adequately represented exposure; *d*) a 2-month average for the pollutants is highly correlated with the annual average, suggesting that different metrics of chronic exposure would not produce significantly different results (data not shown); and *e*) previous analyses have found that using longer windows with declining levels of particulate matter can bias the results (Woodruff et al. 1997).

These exclusions led to a final study population of 3,583,495 births, including 6,639 postneonatal deaths occurring in 96 counties throughout the United States. All of these 96 counties were classified by the Office of Management and Budget as metropolitan counties, which are defined as *a*) central counties with one or more urbanized areas, and *b*) outlying counties that are economically tied to the core counties as measured by work commuting (U.S. Department of Agriculture Economic Research Service 2003).

### Infant outcomes

We obtained *International Classification of Diseases, 10th Revision* (ICD-10) (World Health Organization 1993) codes for the underlying cause of death from the death certificate information included in the linked birth and death records. Respiratory mortality primarily included underlying cause of death codes from Chapter 10, “Diseases of the Respiratory System” (J000-99), plus deaths coded P27.1 [bronchopulmonary dysplasia (BPD)]. SIDS was defined as R95, and “Other ill-defined and unspecified causes of mortality” (referred to in this analysis as “ill-defined”) were defined as R99. In addition, we evaluated all other deaths (any death not classified as respiratory, cardiovascular, SIDS, or ill-defined) as a control category. Finally, we further examined the SIDS and other ill-defined cause of death by evaluating them together. We combined the category of SIDS and ill-defined deaths based on a recent analysis by Malloy and MacDorman (2005), which suggested that during our study period, many SIDS deaths may have been classified as R99.

### Analysis

Because the independence assumption needed for ordinary logistic regression may be violated by inclusion of county-level variables (both pollution exposures and census-level covariates) that can lead to within-county correlation among births, we used logistic regression that incorporated generalized estimating equations (GEE) to estimate the odds ratios (ORs) for all-cause and cause-specific postneonatal mortality by exposure to air pollution (SAS Institute Inc., Cary, NC) (Zeger and Liang 1986). An exchangeable correlation structure was assumed for the GEE models, which is appropriate when there is no time dependence among the births within county and any ordering of the births within county is valid within the data (Hardin and Hilbe 2003).

All air pollution exposures were modeled using a continuous, linear form. We evaluated the appropriateness of a linear form from analysis based on quartiles of exposure, and determined the linear form as a reasonable assumption (data not shown). Several covariates were included in the regression models to obtain adjusted estimates. Maternal characteristics from the birth certificate were maternal race/ethnicity (black, white, Hispanic), marital status, age (< 20 years, 20–34 years, ≥ 35 years), education (< 12 years, 12 years, 13–15 years, and > 15 years), and primiparity (first born). Perinatal research has shown that neighborhood-level socioeconomic status (SES) variables in addition to individual-level covariates from the birth certificate can influence perinatal outcomes (Pearl et al. 2001). To control for potential additional confounding that may not be captured by individual-level variables on the birth certificate, we included county-level poverty and per capita income levels from the U.S. Census in the model (U.S. Census Bureau 2000). We included year and month of birth dummy variables to account for time trend and seasonal effects. Finally, we controlled for region of the country, to account for potential confounding by population and PM composition variation, by classifying infants into one of six U.S. regions (Southern California, Northwest, Southeast, Southwest, Northeast, and Midwest) based on the regions defined in the National Morbidity, Mortality, and Air Pollution Study (Samet et al. 2000).

We calculated adjusted and unadjusted ORs for each pollutant in single-pollutant models for overall postneonatal mortality and for each cause of death. We compared these estimates to ORs for each pollutant calculated from multipollutant models to assess potential confounding of copollutants. However, we did not include both PM_2.5_ and PM_10_ in the same model because PM_2.5_ is a component of PM_10_. All ORs are reported after adjustment for the maternal characteristics and spatial and temporal factors described above. In general, adjusting for all the potential confounders in the model slightly decreased the ORs (≤ 1% decrease; unadjusted data not shown). ORs are also reported for an interquartile range (IQR) increase in the pollutant to help standardize comparisons across pollutants.

## Results

Of the 6,639 infant deaths in our data set, there were 576 deaths from respiratory causes, 1,379 deaths from SIDS, 755 deaths from ill-defined causes, and 3,622 deaths due to other causes. Our study cohort was demographically similar to the eligible births in the United States, though we had a slightly higher percentage of mothers with < 12 years of education and a slightly higher percentage of Hispanic and black mothers (with a complementary decrease in white mothers) (Table 1).

The median pollutant concentrations for survivors and deaths are given in Table 2. The exposure measurements were not highly correlated with each other (Table 3), the strongest being a negative correlation between CO and O_3_ (−0.46), reducing the potential for colinearity in multipollutant models.

We found a statistically significant relationship between PM_10_ and respiratory-related causes of death in the single-pollutant models (Table 4). For a 10-μg/m^3^ increase in PM_10_, rather than the IQR increase shown in Table 4, the odds of respiratory-related death increased 16% [OR = 1.16; 95% confidence interval (CI), 1.06–1.27]. There were elevated, but not statistically significant, relationships between respiratory-related postneonatal mortality and both PM_2.5_ and CO in single-pollutant models (Table 4); there were no associations between respiratory deaths and either O_3_ or SO_2_. The relationship between PM_10_ and respiratory-related postneonatal mortality remained elevated and significant in the multipollutant model (Table 5). No other relationships in the multipollutant model were highly elevated or significant for respiratory-related postneonatal mortality (Table 5).

For SIDS, only O_3_ was associated with a significant increased risk in the single-pollutant models (Table 4). For a 10-ppb increase in average O_3_ levels in the first 2 months of life, the odds of SIDS mortality increased 20% (OR = 1.20; 95% CI, 1.09–1.32). In the multipollutant model, the OR for SIDS and O_3_ was not substantially lower than that found in the single-pollutant model (Table 5). We found elevated ORs between ill-defined cause of death and both PM_2.5_ (OR = 1.15; 95% CI, 0.95–1.38) and PM_10_ (OR = 1.13; 95% CI, 0.99–1.30) (for a 10-μg/m^3^ increase).

In single-pollutant models, there were no relationships between any of the pollutants and other causes of death (the control category) (Table 4). However, there were slightly elevated ORs for PM_10_, PM_2.5_, O_3_ and all causes of death, likely driven by the observed cause-specific relationships between PM and respiratory causes and O_3_ and SIDS (Table 4).

To assess the robustness of the reported findings, we conducted further analyses of the PM_10_ and respiratory postneonatal deaths and O_3_ and SIDS. The relationship between O_3_ and SIDS could be influenced by residual confounding by season, despite controlling for month of birth in the models. SIDS deaths follow a temporal pattern, with the lowest percent of annual SIDS deaths occurring in the spring (19%) and the highest percent occurring in the fall (30%) and winter (25%); O_3_ concentrations are highest in the summer. Therefore, we examined the relationship between SIDS deaths and O_3_ by season of birth (Table 6) and found that the ORs were generally consistent among the seasons, with a slight increase for those babies born in the summer.

We then evaluated the relationship between PM_10_ and BPD (ICD-10 code P2.71). Previous studies in California had identified a higher association between BPD and particulate matter, suggesting that infants with BPD may have particular susceptibility to PM (Ritz et al. 2006; Woodruff et al. 2006) because they are most often born premature and have underlying pulmonary pathology. We found a similar but nonsignificant OR for the 158 deaths coded as BPD (OR = 1.19; 95% CI 0.85–1.65; IQR, multi-pollutant model) compared with all post-neonatal respiratory deaths.

Next, we stratified by birth weight to assess potential increased susceptibility for low-birth-weight babies. For respiratory post-neonatal deaths and PM_10_, we found an OR for normal-birth-weight babies of 1.19 (95% CI, 1.05–1.36; IQR, 241 deaths) and for low-birth-weight babies 1.12 (95% CI, 0.95–1.31; IQR, 335 deaths). We found for O_3_ and SIDS an OR for low-birth-weight babies of 1.33 (95% CI, 0.94–1.88; IQR, 245 deaths) and an OR for normal-birth-weight babies of 1.19 (95% CI, 1.01–1.39; IQR, 1,134 deaths).

We also examined the subset of birth records with complete information on maternal cigarette smoking to assess potential confounding by maternal smoking. As we have reported elsewhere, controlling for maternal smoking in the model had no effect on the parameter estimates for any of the pollutants (Darrow et al. 2006). We also evaluated the effect of removing the variable for region in the model for postneonatal respiratory deaths, under the possibility that region is not a confounding factor. This produced an increased OR for PM_10_ of 1.30 (95% CI, 1.04–1.61) for a 10-μg/m^3^ increase. There were no significant relationships for the other pollutants.

We examined the ORs for increasing quartiles of exposures, and found that infants in the highest quartile of exposure had elevated odds of respiratory mortality, compared with infants in the lowest quartile of exposure both for PM_2.5_, of 1.39 (95% CI, 1.04–1.85), and for PM_10_, 1.31 (95% CI, 1.00–1.71), with weaker responses at the lower exposure levels (data not shown for other quartiles). There was a monotonic increase in odds of SIDS for each quartile of O_3_ exposure compared with the lowest quartile (highest quartile OR = 1.51; 95% CI, 1.17–1.96). No other pollutants had elevated ORs in the highest to lowest quartile comparisons.

Finally, we examined only those deaths that occurred within the first 90 days, which most closely matched our exposure metric of the average over the first 2 months of life. We found for PM_10_ and respiratory-related deaths an adjusted OR of 1.25 (95% CI, 1.06–1.47 for an IQR, 249 deaths). For O_3_ and SIDS the adjusted OR was 1.33 (95% CI, 1.13–1.57 for an IQR, 731 deaths).

## Discussion

A recent review of air pollution and children’s health in Europe by the World Health Organization concluded that “the evidence is sufficient to infer a causal relationship between particulate air pollution and respiratory deaths in the post-neonatal period” (World Health Organization 2005). This study, in conjunction with previous U.S. studies, suggests this statement remains true at PM levels found in the United States.

Our study builds on previous large infant cohort studies by adding additional features to the analysis. We have assessed both sizes of particulates and other air pollutants in a national study. We examined the role of air pollution in SIDS, both over the whole study period and by season; this contemporary analysis is important in light of the changing patterns of SIDS in the last 15 years. We have controlled for additional variables—both individual maternal characteristics and contextual SES variables—although these factors had little effect on the reported associations. The observed relationships between PM_10_ and respiratory postneonatal mortality and O_3_ and SIDS remain relatively robust to model alternatives.

Several analytic decisions could have influenced our study population and results. We evaluated those infants that could be linked to multiple pollutants, rather than focusing on the larger group of infants living in counties with just PM monitors, thus reducing our study sample. Studies comparing populations covered by multiple monitors versus those covered by just PM monitors have found that the relationship between PM and general health and birth weight varies only slightly depending on which population is evaluated (Parker and Woodruff, in press; Parker et al., in press). However, the smaller study size does limit our ability to fully evaluate regional difference, such as from variation in populations or PM composition, and to completely characterize the relationship in other parts of the country. This should be explored in future studies.

In addition, the maternal demographics of our study population, though similar to those for all births, does have a slightly higher proportion of women who are at greater risk of adverse birth outcomes (more women are unmarried, of lower education, and black), and are at higher risk of living in an area of poor air quality (Woodruff et al. 2003). Some residual confounding could be influencing our observed results after we controlled for individual-level and contextual-level SES factors, though accounting for these factors did not significantly alter effect estimates.

A recent study of infant deaths in Southern California found a relationship between CO concentrations in the last 2 weeks of life and respiratory postneonatal mortality (Ritz et al. 2006). However, in our study, we did not observe a relationship between postneonatal respiratory mortality and early-life exposure to CO. This discrepancy may be explained partly by differences in exposure metrics. We use a measure that is larger spatially (county averages vs. the closest monitor in Ritz et al.) and temporally different (we used a more chronic measure, average first 2 months of exposure, and Ritz et al. used a more acute measure, average of the last weeks before death). CO may have a more acute effect, or a more refined geographic scale may be needed to better analyze the potential role of this pollutant.

We examined whether a particular respiratory condition, BPD, might have increased susceptibility to exposure to PM, as suggested by two recent analyses in California (Ritz et al. 2006; Woodruff et al. 2006). The OR for this outcome suggested an increased but nonsignificant risk of mortality for exposure to PM_10_ (data not shown). However, the increased OR for BPD found in this study was not as great as in the studies in California. This could be attributed to regional differences in coding for cause of death or regional variation in PM composition.

There remains uncertainty as to which particles are related to respiratory postneonatal infant mortality. We found that PM_10_, but not PM_2.5_, was associated with respiratory infant mortality, though we found a relationship for the highest quartile of PM_2.5_ exposure. A similar source of pollution may be contributing to both PM_10_ and PM_2.5_ in this study, such as combustion-related sources, because the study area includes only urban counties. Several studies suggest the coarse fraction of PM_10_ (PM 2.5–10 μm in diameter) may be responsible for some of the observed associations between PM_10_ and other health outcomes. Using time-series and case–crossover analyses, Lin et. al. (2002) observed a relationship between asthma hospitalization and coarse PM; no relationship was observed with either PM_10_ or PM_2.5_. A recent review of the health effects of coarse PM concluded that compared with fine PM, coarse particles show as strong or stronger acute effects on asthma, chronic obstructive pulmonary disease, and respiratory hospital admissions (Brunekreef and Forsberg 2005). Furthermore, although short-term mortality appears to be more strongly related to fine PM, coarse PM has been associated with cardiovascular morbidity, such as heart rate variability and various types of cardiovascular disease hospital admissions (Brunekreef and Forsberg 2005; Lipsett et al. 2006). A recent study also reports an association between prenatal exposure to coarse particles and decrease birth weight, with little effect from fine particles (Parker and Woodruff, in press).

The relationship between air pollution and SIDS continues to warrant further study. Given our findings of a relationship between O_3_ and SIDS, we might also expect O_3_ to be related to respiratory postneonatal mortality, because O_3_ is a well-established respiratory irritant (Bell et al. 2004; Hubbell et al. 2005). In addition, a recent study by Triche and colleagues (2006) found ambient measures of O_3_ to be associated with respiratory symptoms in infants, such as wheeze and difficulty breathing. However, neither our study nor a recent Southern California study found O_3_ associated with respiratory postneonatal mortality (Ritz et al. 2006). In addition, studies in Southern California and Canada did not find O_3_ associated with SIDS (Dales et al. 2004; Ritz et al. 2006). Differences in exposure metrics, study design, and study populations could contribute to observed differences. We used the O_3_ averaged over the county for the first 2 months of the infant’s life. Ritz et al. (2006) used a more acute exposure metric based on O_3_ from the nearest monitor averaged over the last 2 months or 2 weeks of the infant’s life. Dales et al. (2004) used a time-series approach to assess acute (daily) exposures to O_3_. Our O_3_ exposure metric could be a proxy for some other air pollutant, though we might expect similar findings for other pollutants and SIDS if this were the case. It is also possible that early life is a more susceptible time period for O_3_ exposure.

Shifts in the diagnostic coding from SIDS to primarily ICD-10 R99—”ill-defined and unspecified causes of mortality”—as found by Malloy and MacDorman (2005) may also influence observed results. We found a stronger relationship between PM_10_ and SIDS + ill-defined causes of death, but the association between O_3_ and SIDS + ill-defined causes of death was lower than for SIDS alone. Finally, the back-to-sleep campaign may also be playing some role in differences between associations between PM and SIDS in the 1990s versus little association with SIDS around the year 2000.

As with most other air pollution epidemiology studies, we rely on outdoor monitors as a proxy for exposures. PM ambient monitors have been found to be a reasonable proxy for personal exposures (Sarnat et al. 2001, 2006). In addition, measurements from ambient monitors for gaseous pollutants have been found to be better surrogates for personal particulate exposures than personal gaseous pollutant exposure (Sarnat et al. 2001, 2006). These findings support using ambient monitors to assess exposures from particulate matter. They also suggest that findings of associations between health outcomes and gaseous pollutants may be attributed partially to ambient concentrations of specific PM components.

The exposures in this study focus on a metric of chronic exposure after the infant’s birth. Exposures that occur prenatally may further enhance susceptibility and increase risk to infant death. Studies suggest that air pollution can increase the risk of adverse perinatal outcomes, including growth restriction and preterm delivery, both of which increase risk of infant mortality (Glinianaia et al. 2004; Huynh et al. 2006; Parker and Woodruff, in press; Salam et al. 2005). Future analysis to evaluate the role of prenatal exposures is warranted to evaluate any potential prenatal contribution

This study builds on previous large infant cohort studies by adding additional features to the analysis. We have controlled for additional variables, both individual maternal characteristics and contextual SES variables, and assessed multiple pollutants, using both linear and categorical forms of exposure. The observed relationships between PM_10_ and respiratory postneonatal mortality and O_3_ and SIDS remain relatively robust to model alternatives.

This study provides further support for PM air pollution as a risk factor for respiratory-related postneonatal infant mortality and suggests that O_3_ may play a role in SIDS.

## Figures and Tables

**Table 1:** Characteristics of births in U.S. population, eligible births, and study sample for singleton infants: United States, 1999–2002.

Maternal factors	Births^a^ (*n* = 16,066,160)	Eligible births^b^ (*n* = 7,991,974)	Study cohort^c^ (*n* = 3,590,134)	Deaths (*n* = 6,639)
Age (mean years)	27.2	27.6	27.3	25.3
Married (%)	66.6	64.5	61.8	39.7
Parity (%)				
First birth	39.8	40.4	39.8	35.1
Missing	0.4	0	0	0
Education [years (%)]				
< 12	21.3	23.5	27.6	38.7
12	31.2	29.7	29.9	34.1
13–15	21.4	20.8	19.9	16.9
> 15	24.6	26.1	22.7	10.3
Missing (%)	1.5	0	0	0
Race/ethnicity (%)				
Black	14.8	18.5	18.8	39.8
Hispanic	20.6	29.9	37.3	29.4
White	58.8	51.6	43.8	30.9
Asian/Pacific Islander	4.8	0	0	0
American Indian	1.0	0	0	0

a Singleton births in United States 1999–2002 excluding those in U.S. territories.

b Eligible infants were born and resided in a county with > 250,000 people; were singletons; had complete information on birth weight, gestational age (≤ 44 weeks), race, maternal education, maternal age, marital status, and parity; and lived for at least 28 days.

c Eligible births that could be linked to PM_2.5_, PM_10_, O_3_, SO_2_, and CO monitoring data were included in this study cohort.

**Table 2:** Median and IQR (25th–75th percentiles) of PM_10_, PM_2.5_, SO_2_, O_3_, and CO concentrations for survivors and specific causes of postneonatal infant death in the study population for 1999–2002.

Population subset	No.	PM_10_ (μg/m^3^)	PM_2.5_ (μg/m^3^)	CO (ppm)	SO_2_ (ppb)	O_3_ (ppb)
Survivors	3,583,495	28.9 (23.3–34.4)	14.8 (11.7–18.7)	0.67 (0.48–0.87)	2.81 (1.64–4.35)	26.6 (19.6–32.2)
All causes of death	6,639	29.1 (23.9–34.5)	14.9 (12.0–18.6)	0.70 (0.48–0.87)	3.14 (1.79–4.83)	26.4 (19.8–32.0)
Respiratory^a^	576	29.8 (24.3–36.5)	14.8 (11.5–18.5)	0.72 (0.52–0.93)	3.01 (1.85–4.63)	25.8 (19.7–31.6)
SIDS^b^	1,379	28.6 (23.5–33.8)	14.5 (12.0–17.5)	0.63 (0.46–0.83)	3.42 (1.92–5.10)	27.4 (20.7–32.7)
SIDS + ill-defined^c^	2,134	28.8 (23.9–33.9)	14.8 (12.1–18.5)	0.64 (0.47–0.85)	3.13 (1.80–4.90)	26.7 (20.0–32.1)
Other causes^d^	3,622	29.2 (23.9–34.5)	14.9 (12.0–18.6)	0.67 (0.49–0.88)	3.17 (1.78–4.79)	26.2 (19.6–32.0)

a ICD-10 codes J000-J984, P271.

b ICD-10 code R95.

c ICD-10 codes R95 and R99.

d ICD-10 code not in respiratory, SIDS, ill-defined or cardiac-related (codes I219-I999) (ICD-10, World Health Organization 1993).

**Table 3:** Spearman correlation coefficients between PM_10_, PM_2.5_, SO_2_, O_3_, and CO concentrations of average first 2 months of exposure for infants born 1999–2002 in the United States.

	PM_10_	PM_2.5_	CO	SO_2_	O_3_
PM_10_	1				
PM_2.5_	0.34	1			
CO	0.18	0.35	1		
SO_2_	0.00	0.21	0.27	1	
O_3_	0.20	−0.10	−0.46	−0.22	1

**Table 4:** Adjusted^a^ ORs for single-pollutant models and selected causes of postneonatal infant mortality in the United States, 1999–2002.

	OR for an IQR increase (95% CI)				
Cause of death	PM_10_ (μg/m^3^) (IQR 11 μg/m^3^)	PM_2.5_ (μg/m^3^) (IQR 7 μg/m^3^)	CO (IQR 0.39 ppm)	SO_2_ (IQR 2.7 ppb)	O_3_ (IQR 12.6 ppb)
All causes	1.04 (0.99–1.10)	1.04 (0.98–1.11)	1.01 (0.95–1.07)	0.96 (0.91–1.02)	1.05 (0.98–1.13)
Respiratory^b^	1.18 (1.06–1.31)	1.11 (0.96–1.29)	1.14 (0.93–1.40)	1.05 (0.94–1.18)	0.92 (0.76–1.10)
SIDS^c^	1.02 (0.89–1.16)	1.01 (0.86–1.20)	0.88 (0.76–1.03)	0.95 (0.86–1.04)	1.28 (1.13–1.46)
Ill-defined + SIDS^d^	1.06 (0.97–1.16)	1.06 (0.97–1.17)	0.93 (0.84–1.02)	0.95 (0.88–1.04)	1.14 (1.04–1.26)
Other causes^e^	1.02 (0.96–1.07)	1.03 (0.96–1.12)	1.02 (0.97–1.07)	0.98 (0.92–1.04)	1.00 (0.92–1.09)

a Adjusted for individual maternal factors [race/ethnicity (black, white, Hispanic), marital status, maternal education (< 12, 12, 13–15, > 15 years), maternal age (< 20, 20–34, > 35 years), primiparity], percentage population below poverty, region, birth month, and birth year.

b ICD-10 codes J000-J984, P271.

c ICD code R95.

d ICD-10 code R99 or R95.

e ICD-10 code not in respiratory, SIDS, ill-defined or cardiac-related (codes I219-I999) (ICD-10, World Health Organization 1993).

**Table 5:** Adjusted^a^ ORs for multipollutant models for selected causes of postneonatal infant mortality in the United States, 1999–2002.

	OR for an IQR increase (95% CI)				
Cause of postneonatal death	PM_10_ (μg/m^3^) (IQR 11 μg/m^3^)	PM_2.5_ (μg/m^3^) (IQR 7 μg/m^3^)	CO (IQR 0.39 ppm)	SO_2_ (IQR 2.7 ppb)	O_3_ (IQR 12.6 ppb)
Respiratory^b^					
PM_10_, CO, O_3_, SO_2_	1.16 (1.04–1.30)		1.02 (0.89–1.15)	1.07 (0.88–1.29)	0.92 (0.77–1.10)
PM_2.5_, CO, O_3_, SO_2_		1.05 (0.89, 1.24)	1.02 (0.90–1.16)	1.11 (0.88–1.39)	0.95 (0.79–1.14)
SIDS^c^					
PM_10_, CO, O_3_, SO_2_	1.02 (0.90–1.16)		0.96 (0.87–1.06)	0.91 (0.78–1.05)	1.24 (1.08–1.44)
PM_2.5_, CO, O_3_, SO_2_		1.04 (0.87, 1.23)	0.96 (0.87–1.06)	0.90 (0.77–1.06)	1.24 (1.07–1.43)

a Adjusted for individual maternal factors [race/ethnicity (black, white, Hispanic), marital status, maternal education (< 12, 12, 13–15, > 15 years), maternal age (< 20, 20–34, > 35 years), primiparity], percentage population below poverty, region, birth month, and birth year.

b ICD-10 codes J000-J984, P271.

c ICD-10 code R95 (ICD-10, World Health Organization 1993).

**Table 6:** Adjusted^a^ ORs for an IQR increase in O_3_ and postneontal infant mortality due to SIDS in the United States by season of birth.

	Dec–Feb	Mar–May	June–Aug	Sept–Nov
OR for 12.6-ppb increase in O_3_ (95% CI)	1.24 (0.73–2.10)	1.21 (0.85–1.70)	1.43 (1.19–1.73)	1.21 (0.84–1.74)
No. of deaths	246	358	449	326

a Adjusted for individual maternal factors [race (black, white, Hispanic), marital status, maternal education (< 12, 12, 13–15, > 15 years), maternal age (< 20, 20–34, > 35 years), primiparity], percentage of population below poverty, region, birth month, and birth year.
